# Internal Standard Addition System for Online Breath Analysis

**DOI:** 10.1021/acs.analchem.4c01924

**Published:** 2024-06-27

**Authors:** Cedric Wüthrich, Timon Käser, Renato Zenobi, Stamatios Giannoukos

**Affiliations:** † Department of Chemistry and Applied Biosciences, ETHZ, Zurich, CH 8093, Switzerland

## Abstract

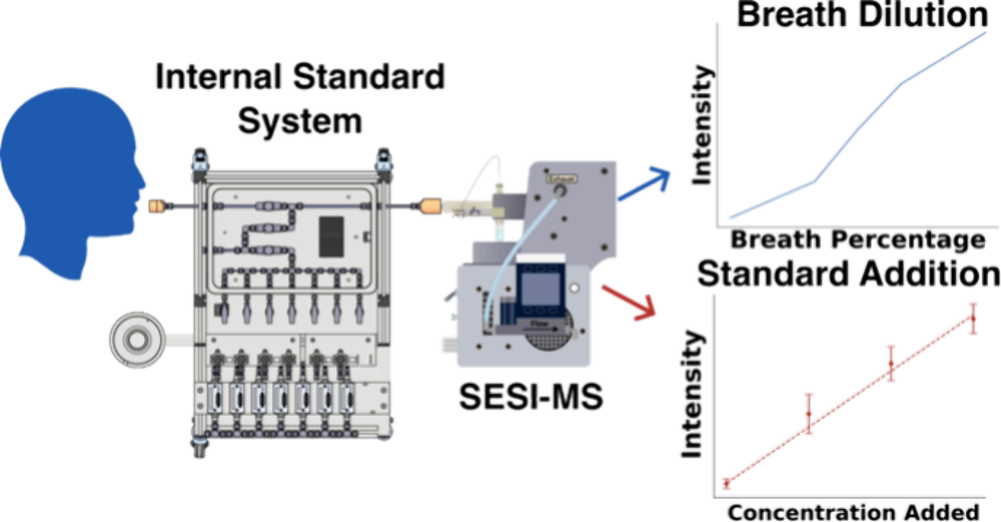

Breath analysis with secondary electrospray ionization (SESI) coupled to mass spectrometry (MS) is a sensitive method for breath metabolomics. To enable quantitative assessments using SESI-MS, a system was developed to introduce controlled amounts of gases into breath samples and carry out standard addition experiments. The system combines gas standard generation through controlled evaporation, humidification, breath dilution, and standard injection with the help of mass-flow controllers. The system can also dilute breath, which affects the signal of the detected components. This response can be used to filter out contaminating compounds in an untargeted metabolomics workflow. The system’s quantitative capabilities have been shown through standard addition of pyridine and butyric acid into breath in real time. This system can improve the quality and robustness of breath data.

## Introduction

Exhaled breath is a valuable biological sample containing volatile and semivolatile organic chemicals (VOCs) partially originating from blood.^1^ The analysis of exhaled breath has the potential to significantly enhance or even replace traditional blood tests, owing to its noninvasive approach. Among breath analysis techniques are online techniques, which can analyze breath without a prior separation stage. They could provide diagnostic information immediately.^2^ There are three major mass spectrometry (MS)-based online techniques for exhaled breath analysis: selected-ion flow tube (SIFT)-MS, proton transfer reaction (PTR)-MS, and secondary electrospray ionization (SESI)-MS. Out of these three techniques, SESI-MS is more adept to analyze heavier and more polar molecules.^2,3^ This makes SESI-MS a compelling technique for breath biomarker discovery. For cystic fibrosis,^4^ obstructive sleep apnea,^5^ chronic obstructive pulmonary disease,^6,7^ and asthma,^8^ explorative and validation studies have been conducted to find and validate biomarkers.

SIFT-MS and PTR-MS, on the other hand, are quantitative techniques. For both, the reaction mechanisms are known, and concentrations can be calculated from the knowledge of kinetic rate constants.^9,10^ The ionization mechanism of SESI is not yet fully understood and remains a topic of ongoing discussion.^11−13^ Consequently, quantitative work with SESI requires reference materials, e.g., gas standards and the generation of calibration curves. Therefore, gas standard delivery systems based on commercial gas bottles^14,15^ and controlled evaporation^16^ for SESI have been developed. These systems can produce gas standards from parts-per-million (ppm) down to parts-per-trillion (ppt) concentrations and can be used for external calibration. However, external calibration in the case of exhaled breath analysis is likely to be inadequate due to ion suppression effects.^17^ The extent of ion suppression in SESI has been shown to be affected by the gas-phase basicity and the concentration of the substances involved. For a complex sample such as breath, with high chemical diversity and varying concentrations,^1^ standard addition emerges as the most appropriate method for quantifying breath components.^18−20^ This method involves the addition of known quantities of analyte to the sample. By linear regression of the analytical response, the concentration of the sample can be calculated. To effectively apply standard addition in exhaled breath analysis with SESI-MS, it is necessary to generate gas standards with concentrations in the range from parts-per-billion (ppb) to ppts and devise a method to introduce these gaseous standards into the breath samples.

The system presented here integrates a gas generation system based on controlled evaporation^16^ with the capability to inject directly into exhaled breath. Besides introducing standards into exhaled breath, the system can also dilute exhaled breath samples. Dilution of breath can potentially lead to an intensity change in the detected signals, facilitating classification of the detected signals: if a signal decreases with increasing dilution, it would likely be part of the exhaled breath and not stem from any contamination in the system. This enables a novel route for distinguishing/filtering features in untargeted breath metabolomics studies. This system offers the unique capabilities to both dilute breath online and inject standard gases into exhaled breath samples.

## Materials and Methods

### Chemicals

Optima LC-MS grade water (Fisher Scientific, USA) was utilized for all aqueous solutions. Formic acid (purity ≥99.99%, Sigma-Aldrich, USA) was dissolved in water with a volume concentration of 0.1% for the sprayed electrolyte solution. For the gas standards, butyric acid (purity ≥99.5%) was obtained from Supelco (USA), D_7_-butyric acid (purity ≥98%) from Cambridge Isotope Laboratories (UK), pyridine (purity ≥99%) from VWR Chemicals (USA) and D_5_-pyridine (purity ≥99.95%) from Acros Organics (Netherlands).

### Standard System

**Figure 1:**
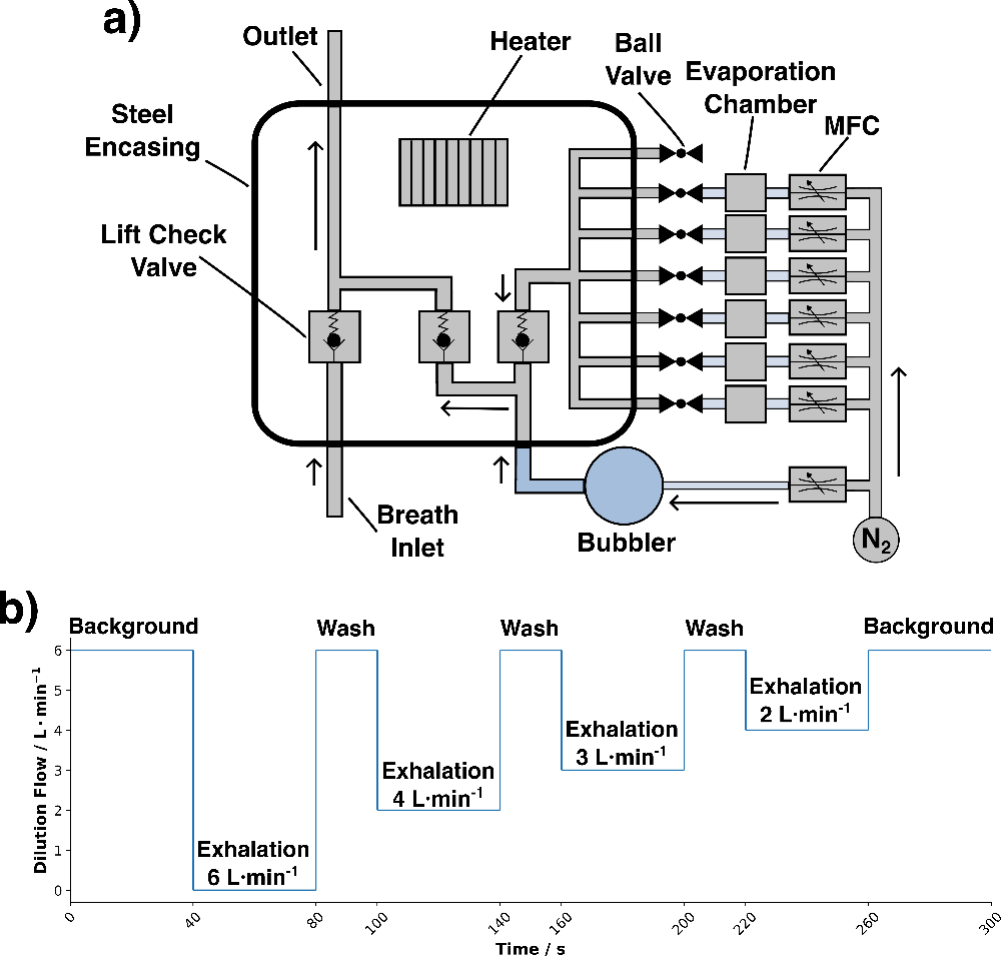
(a) Top-down view of the internal gas standard addition system. Mixing of the different gas streams was conducted in a heated stainless-steel encasement, (b) An example of the procedure for exhaled breath dilution.

The basic principle of the presented system is the dynamic mixing of exhaled breath with a humidified nitrogen stream infused with standard gases. All parts in contact with breath and the gas standard streams were coated with SilcoNert 2000 to prevent adsorption. The system with its mass flow controllers (MFCs) (type GE50A, MKS Instruments, USA) was connected to the in-house nitrogen supply system with the pressure set at 2 bar. All MFCs were arranged in sequence and connected to the in-house nitrogen supply via stainless-steel tubing (with an outer diameter (OD) of 0.25 in, and an inner diameter (ID) of 0.18 in) using union tees (OD: 0.25 in, Swagelok, USA). A single mass flow controller regulated the dilution flow within a range of 1–10 L·min^–1^ and six other MFCs were utilized to regulate flows ranging from 1 to 10 mL·min^–1^ through the evaporation chambers. Within these chambers, gas standards were produced by controlled evaporation at a consistent temperature of 25 °C. The dimensions and operation principles of these chambers were previously reported.^16^ Briefly, an aqueous solution of the desired compound is injected into a chamber. An equilibrium between the solution and gas phase is formed. The gas concentration can be calculated with Henry’s constant. A nitrogen flow further dilutes the gas phase and carries the standard to the instrument. The N_2_ flows passing through the evaporation chambers were regulated by a custom-made LabVIEW (32bit runtime engine 2016, National Instruments, USA) software. The chamber outlets were connected in series to a line of stainless-steel tubing (OD: 0.125 in, ID: 0.085 in) and could be isolated through ball valves (40G, OD: 0.125 in, Swagelok, USA). An additional, unused seventh inlet was kept reserved for potentially connecting other devices for generating gas standards. Gas standard streams flowed through a lift check valve (Swagelok, USA) and was introduced via a union tee into a stainless-steel tube (OD: 0.25 in, ID: 0.18 in) containing the humidified dilution flow. The dilution flow was humidified using a gas-washing bottle (Schott, Germany) filled with water. A stainless-steel mantel heated the bottle and water to 37 °C. The humidified nitrogen flow, spiked with gas standards, was combined with exhaled breath in a union tee. The exhaled breath (OD: 0.25 in, ID: 0.18 in) and the dilution flow tubes were connected to a lift check valve to prevent backflow into the other parts. The final mixed gas stream was directed into the SESI source through an adapter. To minimize condensation, all tubing was housed within a stainless-steel enclosure, which was heated to 60 °C. A schematic illustration of the system is found in Figures 1a, Figure S1a, and Figure S1b.

The procedure illustrated in Figure 1b starts with the dilution flow being adjusted to 6 L/min for 40 s, which serves to establish a baseline measurement of the background. Following this initial phase, there is a 40-s period where exhalation occurs, and this is subsequently followed by a 20-s washing cycle. In this example, the dilution flow is incrementally increased to dilute the exhaled breath at various stages effectively.

### Experimental Set-Up

For measurements involving volunteers, a spirometry filter (Vyaire Medical, Germany) was placed on the breath inlet of the internal gas standard addition system. The standard system was subsequently connected to a SuperSESI ion source (Fossil Ion Tech, Spain) and a flowmeter (EXHALION, Fossil Ion Tech, Spain). The ion source was mounted on a Q-Exactive Plus Orbitrap mass spectrometer (Thermo Fisher, Germany). The sample line of the SESI source was maintained at 130 °C and the ionization chamber at 90 °C. The electrospray was generated by passing an aqueous formic acid solution (0.1% v/v) through a nanoelectrospray capillary (ID: 20 mm, OD: 365 mm, Fossil Ion Tech, Spain) at an overpressure of 0.8 bar, a sheath gas flow of 15 psi, an auxiliary gas flow of 2 arbitrary units (a.u). and an applied voltage of ±3.5 kV. The ion transfer capillary of the mass spectrometer was heated to 250 °C. The automatic gain control (AGC) value of the Orbitrap was set to 10^6^, the mass resolution to 140’000 at 200 m/*z*, and a maximum inject time of 500 ms. The set windows for each of the different experiments are shown in Table S1.

### Dilution and Addition Experiments

To assess the dilution performance of the internal gas standard addition system, the breath of volunteers was diluted with different air flows. The target was to combine each exhalation with the dilution flow and to achieve a total flow of 6 L·min^–1^. The dilution flow was set to 0, 2, 3, and 4 L·min^–1^ in 40 s intervals. During these intervals, volunteers provided one exhalation (∼30 s) to reach the target flow rate. To measure a baseline, a period at the beginning and end of the procedure was recorded with a constant dilution flow at 6 L·min^–1^. An example of the dilution flow rate is illustrated in Figure 1b. For each volunteer, three sets of measurements in each ion mode (positive and negative) were recorded: one with increasing dilution levels, one with decreasing dilution levels, and one with a mixed sequence order of dilution flows (3, 0, 4, 2 L·min^–1^).

To demonstrate the internal gas standard system mixing capabilities with exhaled breath, a standard addition experiment was conducted. This included three volunteers (Ethics waiver: EK-2024-E-2) and included the controlled addition of pyridine and butyric acid into the exhaled breath samples for quantification (detected *m*/*z* values in Table S2). For the addition of gaseous standards into exhaled breath, the dilution flow was set to 2 L·min^–1^, necessitating a 4 L·min^–1^ of breath flow from each volunteer. For the standard addition experiments, the concentration of the gas standards was increased by increasing the flow through the evaporation chambers from 0 to 8 mL·min^–1^ in increments of 2 mL·min^–1^. For pyridine and butyric acid, the concentration added was increased in discrete steps from 7 up to 30 ppb’s (detailed concentrations in Table S3–S4). The deuterated analogs of these compounds were added at a constant concentration as internal standards. Specifically, 7 ppb of D_5_-pyridine was added to breath with a 2 mL·min^–1^ flow rate through the evaporation chamber.

For optimal operation, the evaporation chambers were charged with approximately 10 μL of the aqueous stock solution before each individual measurement.

The humidity and temperature at the system outlet were measured with an FH A646 R ALMEMO 2590-2A/-4AS humidity sensor (Ahlborn Mess-and Regelungstechnik GmbH, Germany).

### Data Processing and Analysis

Mass spectra in the RAW format were converted into the mzML format^21^ with ProteoWizard^22^ and processed with a custom Python (v3.11) script afterward. The script averaged the obtained mass spectra and detected peaks in the obtained spectrum using a height filter of 10^3^. Subsequently, time traces were obtained through the integration of the individual peaks within each scan of all measurements. The traces were then aligned with the corresponding flow profiles. Average signal intensities were then obtained by averaging the signal during exhalations. For the standard gases, their corresponding [M + H]^+^ intensities were used for the linear regression.

Clustering of the feature traces over different dilutions was conducted with bisecting K means implemented in the scikit-learn (v1.3) library.^23^ Linear regression of signal intensities was performed with the SciPy (v 1.11) library.^24^

## Results and Discussion

### Dilution of Breath

**Figure 2:**
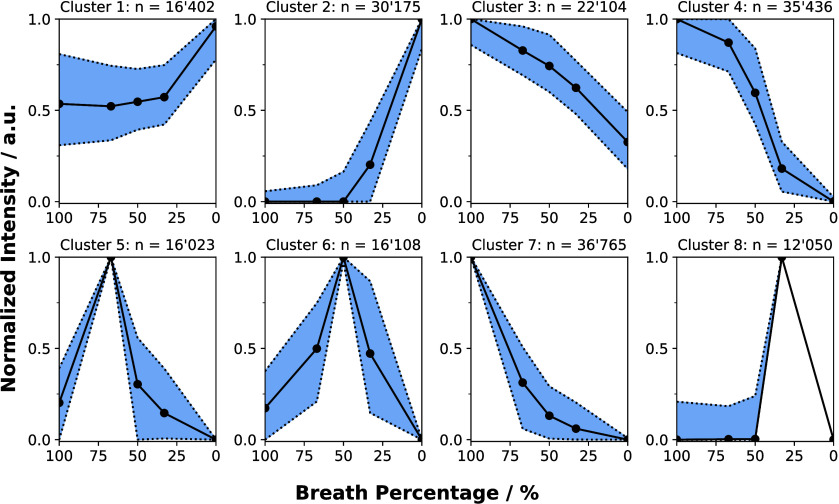
Median normalized intensities of the features clustered according to their behavior over different dilution ratios. The intensity is traced over several dilution ratios, with both at the beginning and end having only the intensity observed during the background measurements. The median is depicted as a black line, and the 25th and 75th percentiles are given as dotted lines. Clusters 1 and 2 contain features, which react negatively when exhaled, linking them probably to background contamination. Clusters 3, 4, and 7 exhibit the highest intensity when only the exhaled air is measured with a subsequent decay of intensity with more dilution. Features in clusters 5, 6, and 8 show increased signal strength when diluted. A possible reason could be the reduction of ion suppression due to dilution.

A significant challenge in SESI-breath analysis so far has been distinguishing features originating from exhaled breath from those caused by background contaminations. This was partly due to the absence of a proper baseline to compare signal intensities. The system introduced here addresses this by enabling the comparison of features against a control of blank humidified nitrogen at the same flow rate as that of exhaled breath and by allowing for the examination of how breath feature intensities change with increasing levels of dilution. To assess the impact of dilution on the signals of breath features, the breath of volunteers was progressively diluted across four exhalation cycles. The humidity was measured to be in the range from 38 to 41% relative humidity at 50 °C at the system’s outlet, therefore excluding the humidity’s influence on signal strength. This was independent of the chosen dilution factor. Continuous observation of the humidity with the system connected to the ion source was not possible since condensation occurred on the sensor when placed into the gas stream. To visualize the results, the corresponding feature intensities were clustered with bisecting K-means (Figure 2). A representation of the individual signal traces can be found in Figure S2.

Analysis of dilution effects within the data clusters revealed three distinct patterns (Figure 2). As visible through the median of intensity in clusters 1 and 2, some features decrease in intensity upon exhalation compared to when no exhalation occurs. This trend suggests these features were primarily associated with background contaminants—constituting about a quarter of all detected features. In a data preprocessing workflow for untargeted metabolomics, these features could be filtered out. Conversely, clusters 3, 4, and 7 exhibited decreasing intensity with increasing dilution, with the most pronounced effects when no dilution was applied. This would be the expected signal behavior upon increased dilution. Features in cluster 3 distinguish themselves by already having a nonzero intensity in the background. It could be that the compounds behind these features are already present in the background. Alternatively, these features could be two different constitutional isomers, one in the background and one in exhaled breath. Cluster 4 displayed a proportional decrease in intensity with dilution, expected from linear dilution effects. The exception in this trend is the change from only measuring breath at 6 L·min^–1^ to diluting breath with 2 L·min^–1^ of nitrogen. This nonlinearity of the feature intensity likely stems from inconsistent exhalations at a flow of 6 L·min^–1^, since the system has some internal resistance due to the lift check valves. In cluster 7, feature intensities decreased exponentially with dilution.

Clusters 5, 6, and 8 presented particularly interesting behaviors, with feature intensities peaking during dilution. This could be attributed to reduced ion suppression from dilution potentially enhancing the detectability of previously suppressed compounds.^17,25^ It has been previously shown that an increased concentration of a basic compound in the gas phase can suppress the signal of others.^17^ Through dilution, the effective concentration of basic compounds in exhaled breath was lowered potentially leading to a signal gain for less basic compounds.

This method of assessing feature intensities in response to dilution significantly enhances the ability to filter and refine features for chemometric analysis, markedly improving data quality. An example of such a filtering workflow is shown in Figure S3.

### Standard Addition

**Figure 3:**
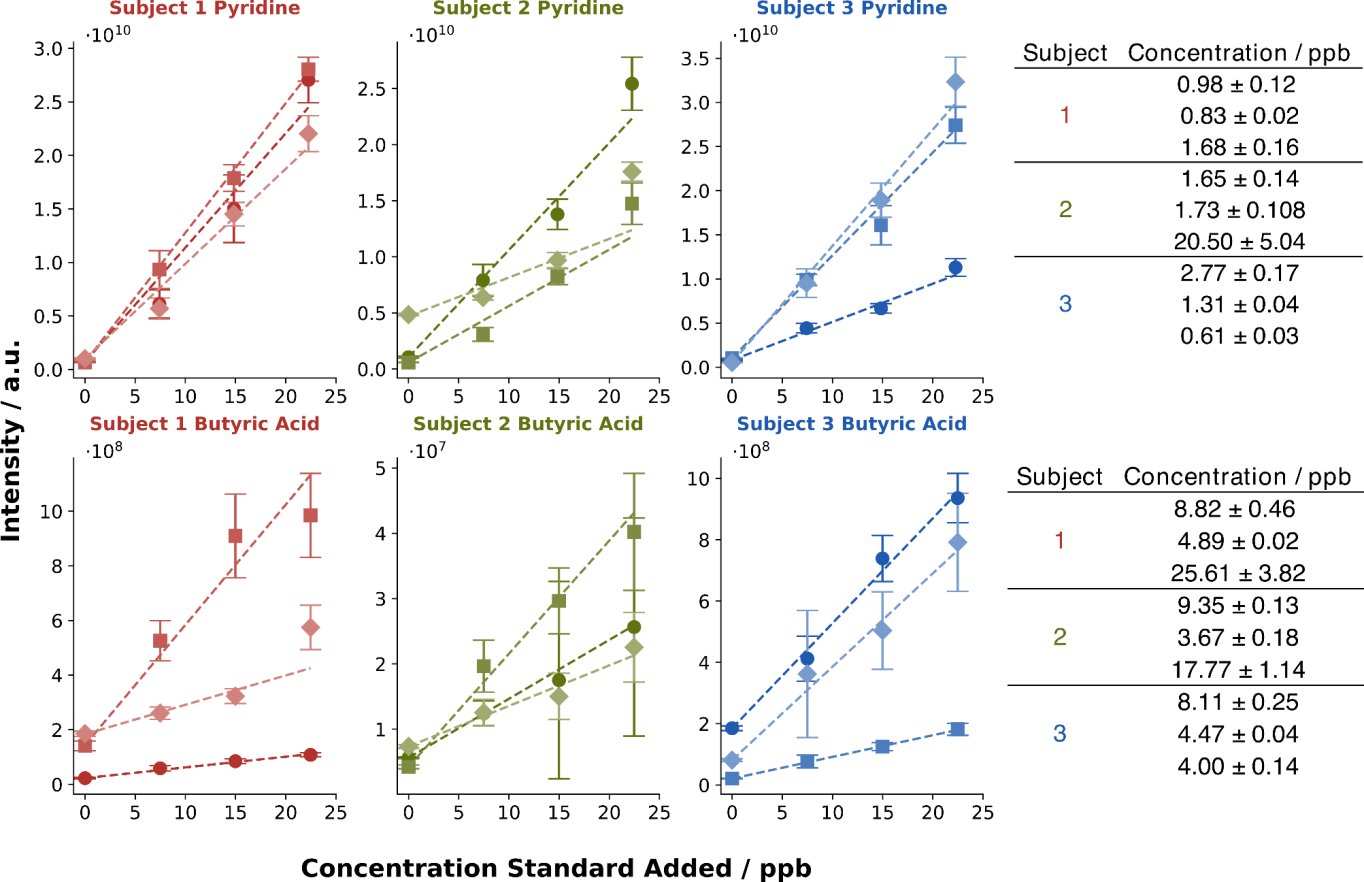
Individual calibration curves for standard addition of pyridine and butyric acid. The standard addition was performed at three different time points for three subjects. The determined concentrations are depicted on the right side.

Due to ion suppression, which compromises the accuracy of quantifying breath components via external calibration, standard addition emerges as the most effective method for quantitative analysis using SESI-MS. The system described herein is capable of producing gas standards of low concentration, with the potential for concentration enhancement facilitated by the mass flow controllers. With multiple evaporation chambers attached to the system, it is possible to obtain calibration curves for multiple compounds simultaneously. To demonstrate this capability, standard addition experiments were performed with pyridine and butyric acid. Three subjects provided exhalations on three separate occasions. In five exhalations, the standards were spiked into breath with increased concentration. Through the obtained signal intensities ([M + H]^+^), a linear regression was drawn, and the breath concentration was determined. The highest added concentration was excluded from the regression (Figure S4) since, in some measurements, the signal deviated from linearity. All fit parameters of the linear regression can be found in Tables S5–S10. The calibration curves obtained and breath concentrations are depicted in Figure 3.

The standard addition curves obtained displayed variability in slopes both within individual subjects and across different subjects. Yet, the calculated concentrations largely fell within the same order of magnitude, with only a few exceptions. This suggests variations in ionization efficiency from one measurement to another. The variation between different measurements could have a biological component. The change in the breath composition might not only affect the intensity itself but also its variation. To assess these variations, four calibration curves were recorded over 4 days at the same concentration levels as in the standard addition experiments. By comparison of the slopes (Figure S5 and Figure S6), an estimate of ion suppression could be performed for pyridine and butyric acid. The sensitivity of pyridine decreased by 21% and of butyric acid by 84% during an exhalation. Comparison of sensitivities also allowed for the estimation of biological and technical variation for the two recorded compounds by comparison of the coefficients of variation between standard addition and external calibration. For pyridine, standard addition gave a CV of 2.5% and external calibration of 2.3%, whereas butyric acid had CVs of 5.0% and 4.7% respectively. Assuming that the technical variation originating from the standard system, the ion source, and the mass spectrometer are the same between the standard addition experiments and the external calibration, the biological variation accounts for less than 10% of the total variation. Normalization of the signals of pyridine and butyric acid with their isotopologues added at a constant concentration worsened the linearity of the calibration curves. Concentration calculation was, therefore, not conducted with the normalized data.

The variation of the signal’s standard deviation was not the same with increased concentration. This could partially be attributed to the fluctuation of the mass flow controller with higher flow rates. Another factor could be the altered composition and humidity level of exhaled breath, which would affect signal stability. As previously discussed, it is difficult to measure the humidity of exhaled breath directly in its path, and thus the factor of humidity remains uncontrolled. The same effects were probably responsible for the outliers observed in the standard addition data.

The system outlined here simplifies standard addition experiments by enabling automated concentration adjustments via mass-flow controllers, which regulate the dilution gas flow through evaporation chambers. Consequently, any compound with a sufficiently low *Henry’s* constant can be spiked into breath and quantified. For compounds that are not soluble in water, it could be possible to switch to an alternate solvent such as acetonitrile. The drawbacks would be the absence of tabulated constants for liquid–gas phase equilibria and the potential alteration of sensitivity with increased levels of solvents that are not water. It is even possible to enhance the system further by connecting an aerosol generator to the inlet.

## Conclusions

SESI-MS-based breath analysis is not a quantitative technique as the ionization mechanism is not known. To enhance quantification capabilities, a system was developed to spike breath with precise amounts of gaseous standards. This system not only facilitates quantification through standard addition, but also serves to dilute breath. Dilution is particularly beneficial in untargeted metabolomics studies, because it aids in the more effective discrimination of metabolic features, thereby enhancing the overall quality of the data. In principle, the system could be connected to aerosol generators, thus allowing for the spiking of breath with nonvolatile compounds. The inclusion of such a system in any breath-related study could significantly improve the robustness of the results.

## Data Availability

The original data used in this publication are made available in a curated data archive at ETH Zürich (https://www.research-collection.ethz.ch) under the DOI: 10.3929/ethz-b-000667769.

## Supporting Information Available

Tables reporting the *m*/*z* window values, Henry’s constants for the used compounds, concentrations in the gas phase, and fit parameters of all linear regression. Additional figures showing the standard addition system. Plots of the slopes of the calibration and standard addition curves. (PDF)
