# Supplementary material for: Internal Standard Addition System for Online Breath Analysis
Source: Anal Chem. 2024 Jun 27;96(27):10871–6. doi: 10.1021/acs.analchem.4c01924 (PMC11238155; doi:10.1021/acs.analchem.4c01924)
Supplement: Supplementary file 1 — ac4c01924_si_001.pdf [file ac4c01924_si_001.pdf]

Supporting Information

Internal Standard Addition System for On-Line Breath Analysis

Cedric Wüthrich[1], Timon Käser[1], Renato Zenobi[1*], Stamatios Giannoukos[1*]

(1) Department of Chemistry and Applied Biosciences, ETHZ, Zurich, CH-8093, Switzerland

Correspondence: Stamatios Giannoukos (stamatios.giannoukos@org.chem.ethz.ch), Renato Zenobi (renato.zenobi@org.chem.ethz.ch)

Table of contents

Label	Content	Page
Table S1	Acquisition Parameters	S3
Table S2	*m/z* values of the standards	S4
Table S3	*Henry's* constants	S5
Table S4	Gas concentrations	S6
Table S5	Fit parameters of linear regression of the butyric acid standard addition without the last point	S7
Table S6	Fit parameters of linear regression of the butyric acid standard addition including the last point	S8
Table S7	Fit parameters of linear regression of the pyridine standard addition without the last point	S9
Table S8	Fit parameters of linear regression of the pyridine standard addition including the last point	S10
Table S9	Butyric acid calibration curve fits	S11
Table S10	Pyridine calibration curve fits	S12
Figure S1	Schematics of the standard addition system	S13
Figure S2	Feature intensity clustering with the individual features visible	S14
Figure S3	Feature filtering workflow	S15
Figure S4	Standard addition curves with last point included	S16
Figure S5	Slopes of the standard addition curves	S17
Figure S6	Boxplot of the different slopes	S18
References		S18

Table S1. Chosen window sizes for the acquisition with the *Orbitrap Q-Exactive*. For positive and negative ion mode measurements for the breath dilution experiments, the windows were chosen according to literature.[1]

Experiment	Window 1	Window 2	Window 3	Window 4	Window 5
Breath Dilution Positive Ion Mode	50-500 *m/z*	50-135 *m/z*	132-195 *m/z*	195-277 *m/z*	277-500 *m/z*
Breath Dilution Negative Ion Mode	50-500 *m/z*	50-90 *m/z*	90-161 *m/z*	161-255 *m/z*	255-500 *m/z*
Standard Addition / External Calibration	50-500 *m/z*	79.5-80.5 *m/z*	84.5-85.5 *m/z*	88.5-90.5 *m/z*	95.5-96.5 *m/z*

Table S2. Exact and detected *m/z* values of the utilized gas standards.

Standard	Exact *m/z* $[M+H]^+$	Detected *m/z* $[M+H]^+$	Δ /ppm
Pyridine	80.0495	80.0494	-1.25
D5-Pyridine	85.0809	85.0808	-1.18
Butyric Acid	89.0597	89.0596	-1.12
D7-Butyric Acid	96.1036	96.1035	-1.04

Table S3. *Henry's* constants[2] used for the used standards and the concentrations of the solutions injected into the evaporation chambers. For the deuterated analoga, the same constants as the non-deuterated compounds were taken.

Standard	*Henry's* Constant / $mol\cdot m^{-3}\cdot Pa^{-1}$	Solution Concentration / $10^{-4}\cdot M$
Pyridine	1.1	9.10
D5-Pyridine	1.1	8.52
Butyric Acid	47	9.19
D7-Butyric Acid	47	9.09

Table S4. Gas concentrations, which were added in the standard addition experiments listed in increasing order.

Standard	c1 / ppb	c2 / ppb	c3 / ppb	c4 / ppb
Pyridine	7.42	14.83	22.24	29.64
D_5-Pyridine	6.95	13.89	20.82	27.76
Butyric Acid	7.49	14.97	22.45	29.93
D_7-Butyric Acid	7.40	14.80	22.20	29.58

Table S5. Fit parameters of the linear regression (a*concentration+b) and the corresponding fit parameters for butyric acid standard addition. The final calculated concentration is given as c.

Subject	a	a Error	b	b Error	R^2	c/ ppb	c Error / ppb
1	3.91E+06	1.88E+05	2.30E+07	1.44E+06	0.98	8.82	0.46
1	4.40E+06	5.01E+05	1.43E+07	1.76E+06	0.92	4.89	0.54
1	1.08E+06	2.28E+05	1.84E+07	1.34E+06	0.73	25.61	3.82
2	9.03E+05	1.87E+04	5.63E+06	9.23E+03	0.99	9.35	0.13
2	1.73E+06	1.16E+05	4.24E+06	1.43E+05	0.98	3.67	0.18
2	6.21E+05	5.91E+04	7.35E+06	1.14E+05	0.96	17.77	1.14
3	3.43E+06	1.43E+05	1.85E+07	3.89E+05	0.99	8.11	0.25
3	7.07E+06	8.97E+04	2.11E+07	1.33E+05	1.00	4.47	0.04
3	3.05E+06	1.52E+05	8.13E+06	1.40E+05	0.98	4.00	0.14

Table S6. Fit parameters of the linear regression (a*concentration+b) and the corresponding fit parameters for butyric acid standard addition including the last concentration added. The final calculated concentration is given as c.

Subject	a	a Error	b	b Error	R^2	c/ ppb	c Error / ppb
1	3.39E+05	6.50E+05	2.78E+07	1.63E+07	-0.79	122.66	0.46
1	4.36E+06	3.98E+05	1.44E+07	1.46E+06	0.94	4.94	0.54
1	2.16E+06	7.90E+05	1.65E+07	5.88E+06	0.69	11.48	3.82
2	6.92E+05	9.94E+04	5.63E+06	7.77E+04	0.83	12.20	0.13
2	1.63E+06	7.75E+04	4.25E+06	1.49E+05	0.99	3.90	0.18
2	1.01E+06	1.74E+05	7.29E+06	5.49E+05	0.81	10.85	1.14
3	2.84E+06	1.51E+05	1.86E+07	1.05E+06	0.94	9.84	0.25
3	6.80E+06	1.09E+05	2.11E+07	2.95E+05	1.00	4.65	0.04
3	2.88E+06	1.33E+05	8.13E+06	1.71E+05	0.97	4.24	0.14

Table S7. Fit parameters of the linear regression (a*concentration+b) and the corresponding fit parameters for pyridine standard addition. The final calculated concentration is given as c.

Subject	a	a Error	b	b Error	R^2	c/ ppb	c Error / ppb
1	1.07E+09	1.27E+08	7.00E+08	1.04E+08	0.96	0.98	0.12
1	1.21E+09	2.22E+07	6.70E+08	2.04E+07	1.00	0.83	0.02
1	8.88E+08	7.27E+07	9.96E+08	1.21E+08	0.98	1.68	0.16
2	9.55E+08	7.84E+07	1.05E+09	1.03E+08	0.96	1.65	0.14
2	5.03E+08	6.87E+07	5.81E+08	4.37E+07	0.91	1.73	0.18
2	3.45E+07	1.21E+07	4.71E+08	5.35E+07	0.71	20.50	5.04
3	4.33E+08	2.91E+07	7.99E+08	5.07E+07	0.98	2.77	0.17
3	1.17E+09	4.51E+07	1.01E+09	3.04E+07	0.99	1.31	0.04
3	1.32E+09	6.97E+07	5.36E+08	2.51E+07	0.99	0.61	0.03

Table S8. Fit parameters of the linear regression (a*concentration+b) and the corresponding fit parameters for pyridine standard addition including the last concentration added. The final calculated concentration is given as c.

Subject	a	a Error	b	b Error	R^2	c/ ppb	c Error / ppb
1	1.00E+09	4.22E+07	7.01E+08	9.09E+07	0.97	1.05	0.12
1	1.24E+09	1.48E+07	6.69E+08	2.39E+07	1.00	0.81	0.02
1	9.82E+08	1.10E+08	9.87E+08	2.10E+08	0.90	1.51	0.16
2	9.73E+08	7.12E+07	1.05E+09	9.72E+07	0.93	1.62	0.14
2	5.92E+08	1.14E+08	5.80E+08	8.11E+07	0.77	1.47	0.18
2	3.68E+07	1.24E+07	4.68E+08	5.58E+07	0.30	19.08	5.04
3	4.61E+08	3.74E+07	7.96E+08	7.36E+07	0.94	2.59	0.17
3	1.24E+09	3.11E+07	1.01E+09	4.20E+07	0.98	1.22	0.04
3	1.30E+09	4.80E+07	5.36E+08	2.18E+07	0.99	0.62	0.03

Table S9. Parameters for the linear regression of the calibration curves (a*concentration+b) for butyric acid.

Index	a	a Error	b	b Error	R^2
0	5.49E+06	2.31E+07	1.72E+06	2.71E+07	0.63
1	3.30E+06	2.58E+07	1.18E+06	2.24E+07	0.78
2	8.35E+06	-4.06E+07	1.72E+06	2.24E+07	0.93
3	7.88E+06	-1.72E+07	1.80E+05	3.82E+06	0.99
4	5.63E+06	1.98E+07	3.70E+05	6.00E+06	0.99
5	1.62E+07	-2.02E+07	1.27E+06	1.95E+07	0.97
6	1.40E+07	1.05E+08	2.16E+06	3.14E+07	0.92
7	1.74E+07	3.26E+05	8.80E+05	1.09E+07	0.99
8	1.35E+07	2.04E+07	7.87E+05	1.16E+07	0.99
9	1.36E+07	-2.74E+07	4.13E+06	4.37E+07	0.85
10	1.26E+07	2.83E+07	7.41E+06	9.59E+07	0.66
11	2.95E+07	-1.03E+08	2.88E+06	3.11E+07	0.98
12	1.19E+07	4.26E+07	2.54E+06	2.77E+07	0.91
13	1.90E+07	-2.25E+07	9.72E+05	1.06E+07	0.99
14	2.59E+07	-5.60E+07	3.56E+06	4.24E+07	0.97
15	2.82E+07	-7.02E+07	1.89E+06	2.11E+07	0.98

Table S10. Parameters for the linear regression of the calibration curves (a*concentration+b) for pyridine.

Index	a	a Error	b	b Error	R^2
0	5.49E+06	2.31E+07	1.72E+06	2.71E+07	0.63
1	3.30E+06	2.58E+07	1.18E+06	2.24E+07	0.78
2	8.35E+06	-4.06E+07	1.72E+06	2.24E+07	0.93
3	7.88E+06	-1.72E+07	1.80E+05	3.82E+06	0.99
4	5.63E+06	1.98E+07	3.70E+05	6.00E+06	0.99
5	1.62E+07	-2.02E+07	1.27E+06	1.95E+07	0.97
6	1.40E+07	1.05E+08	2.16E+06	3.14E+07	0.92
7	1.74E+07	3.26E+05	8.80E+05	1.09E+07	0.99
8	1.35E+07	2.04E+07	7.87E+05	1.16E+07	0.99
9	1.36E+07	-2.74E+07	4.13E+06	4.37E+07	0.85
10	1.26E+07	2.83E+07	7.41E+06	9.59E+07	0.66
11	2.95E+07	-1.03E+08	2.88E+06	3.11E+07	0.98
12	1.19E+07	4.26E+07	2.54E+06	2.77E+07	0.91
13	1.90E+07	-2.25E+07	9.72E+05	1.06E+07	0.99
14	2.59E+07	-5.60E+07	3.56E+06	4.24E+07	0.97
15	2.82E+07	-7.02E+07	1.89E+06	2.11E+07	0.98

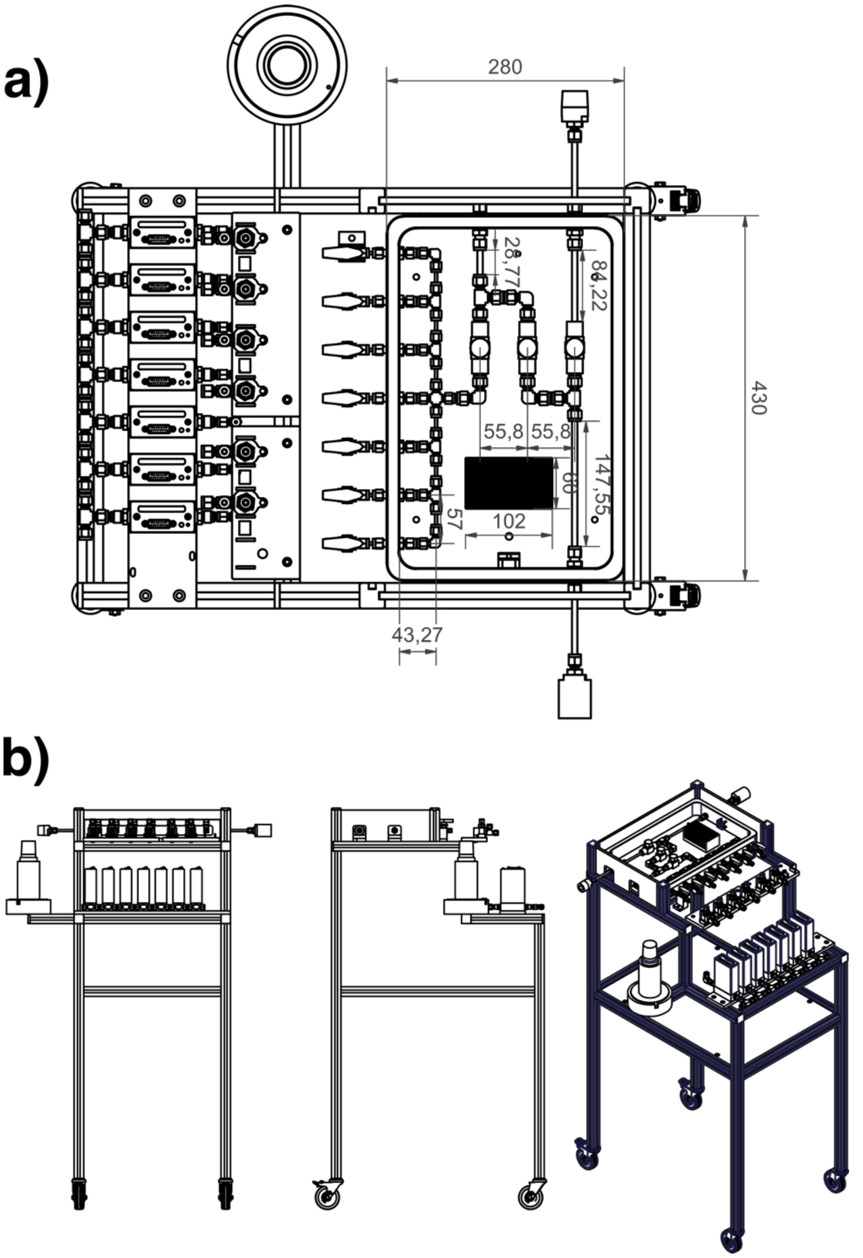

Figure S1. a) Representation of Figure 1a with selected distances inserted. The distances are in mm. The tubing is omitted for clarity. b) Different side-way views of the system.

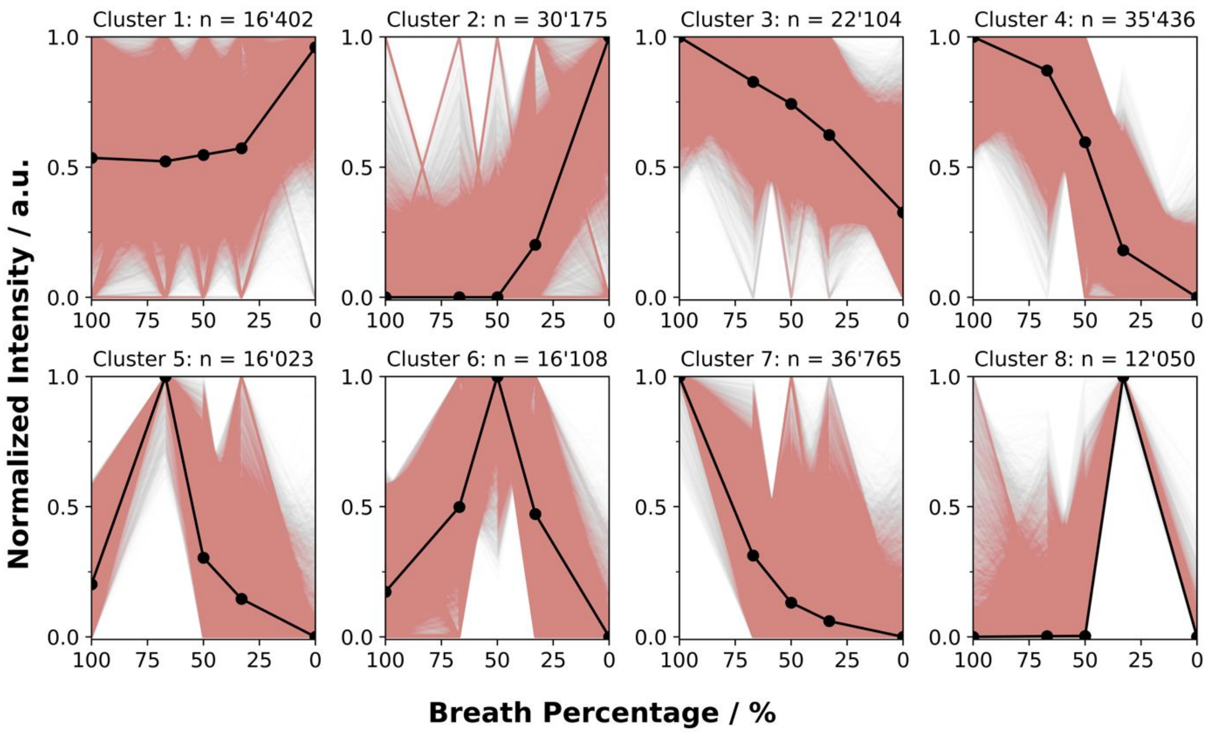

Figure S2. Feature traces (red) and their reaction to dilution are plotted within their respective cluster. The median of all traces within a cluster is depicted as a black line. One feature trace corresponds to the average trace of one subject.

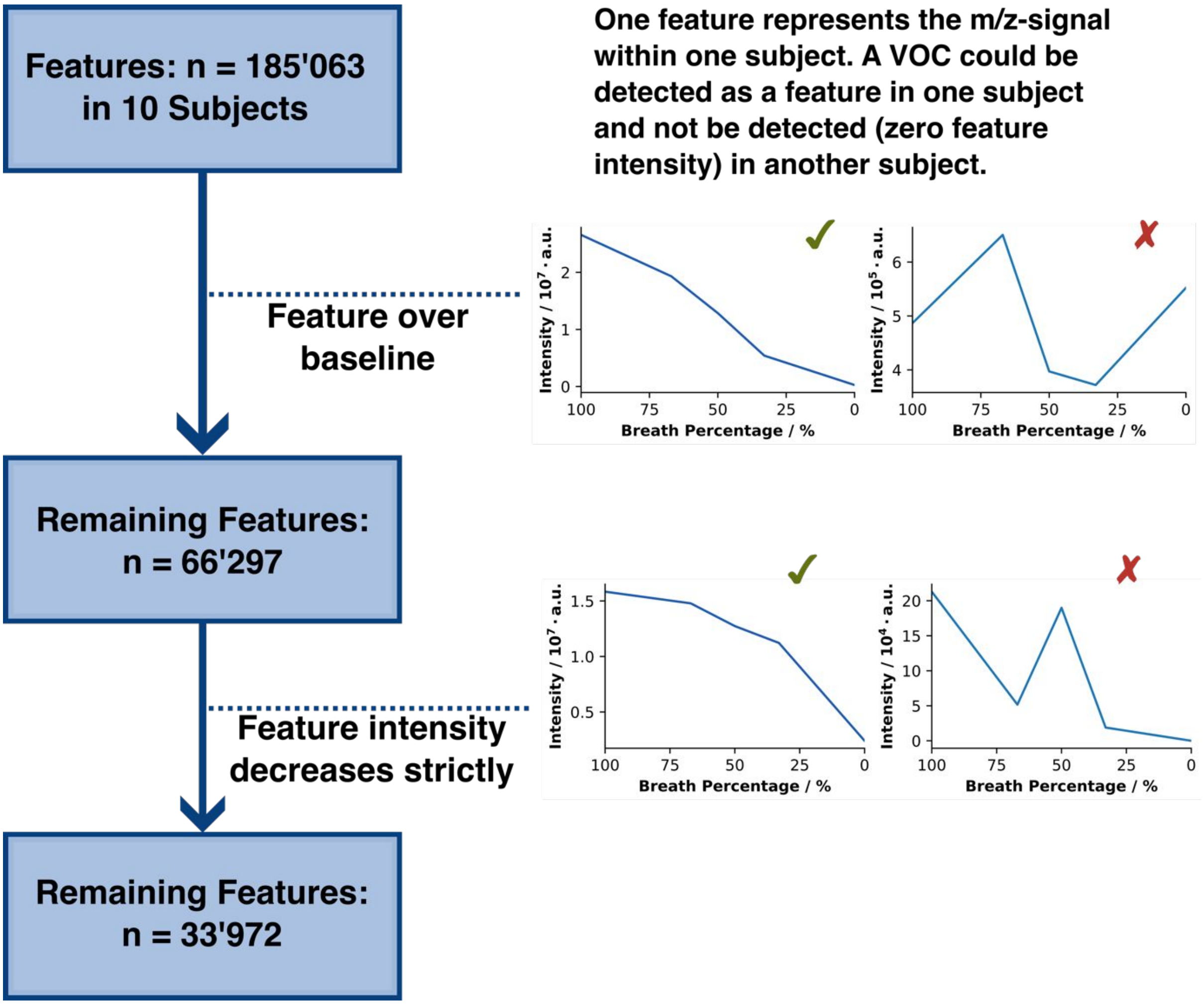

Figure S3. Example feature filtering workflow exploiting the dilution capabilities of the system. The first step involves the removal of features that possess higher intensities during the nitrogen flow set at 6 L·min^{-1}. This nitrogen flow is seen as the baseline. The second step involves the removal of features whose intensities behave atypically with increasing dilution. If a feature's intensity does not fall with increasing dilution, it can be removed. This step does not consider features, which could increase in intensity due to reduced ion suppression (Figure 2).

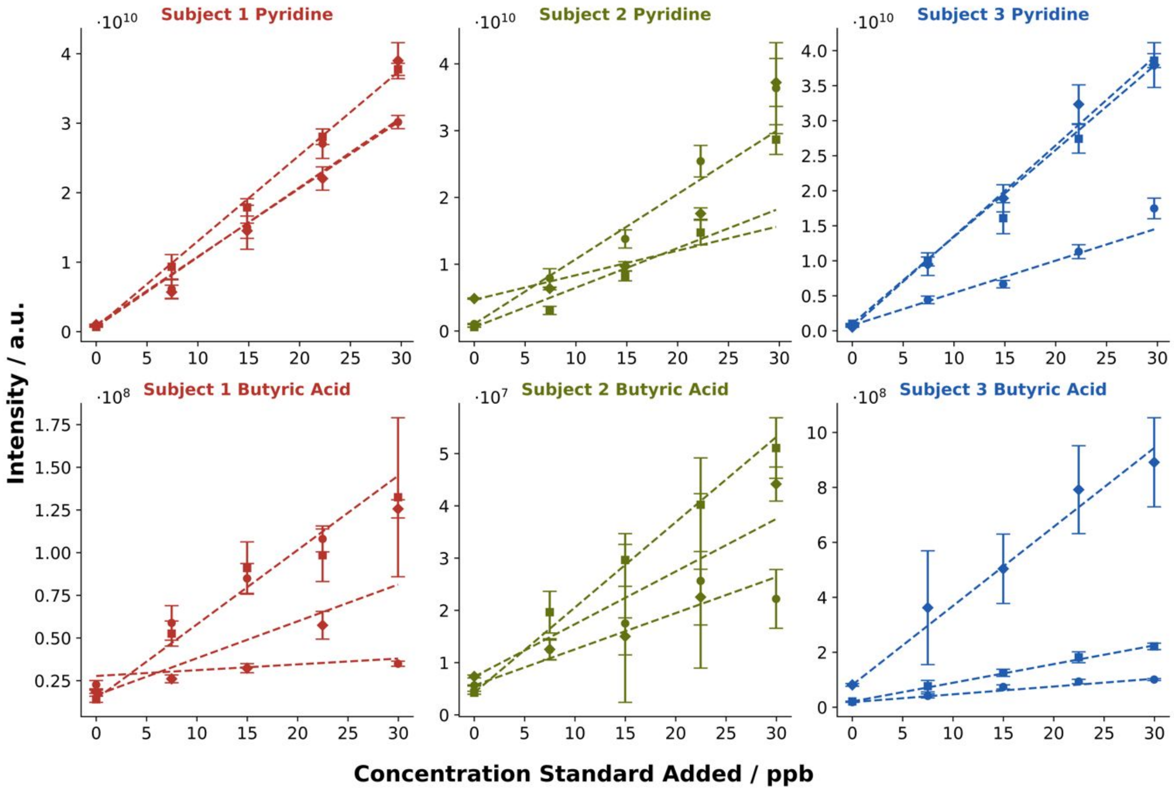

Figure S4. Standard addition curves recorded for pyridine and butyric acid with three subjects. The data points with the highest concentration were excluded from the linear fit depicted in Figure 3 due to the outliers observed.

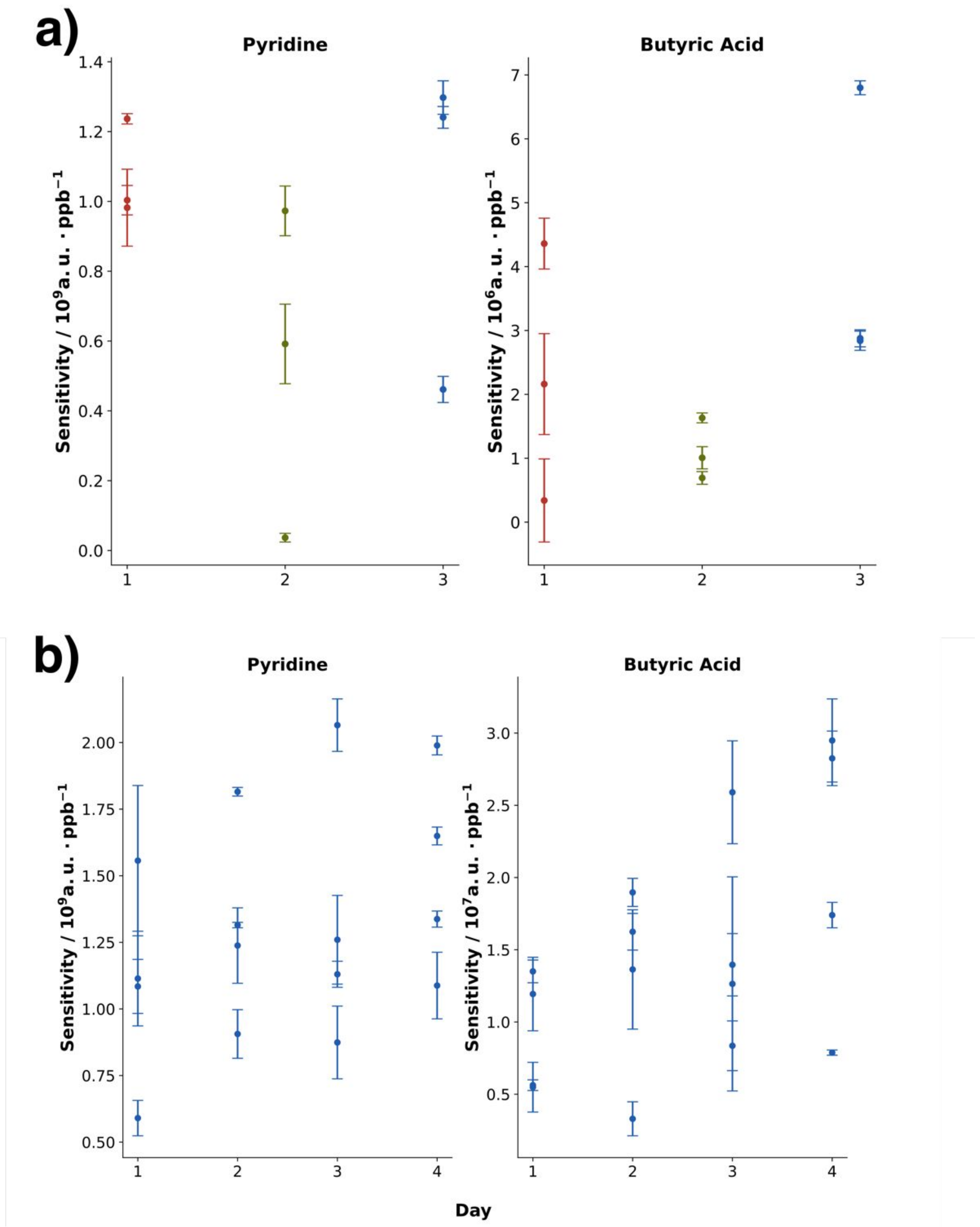

Figure S5. a) Slopes of the standard addition curves according to subject. b) Calibration curve slopes obtained on different days. The slopes are given with their uncertainty.

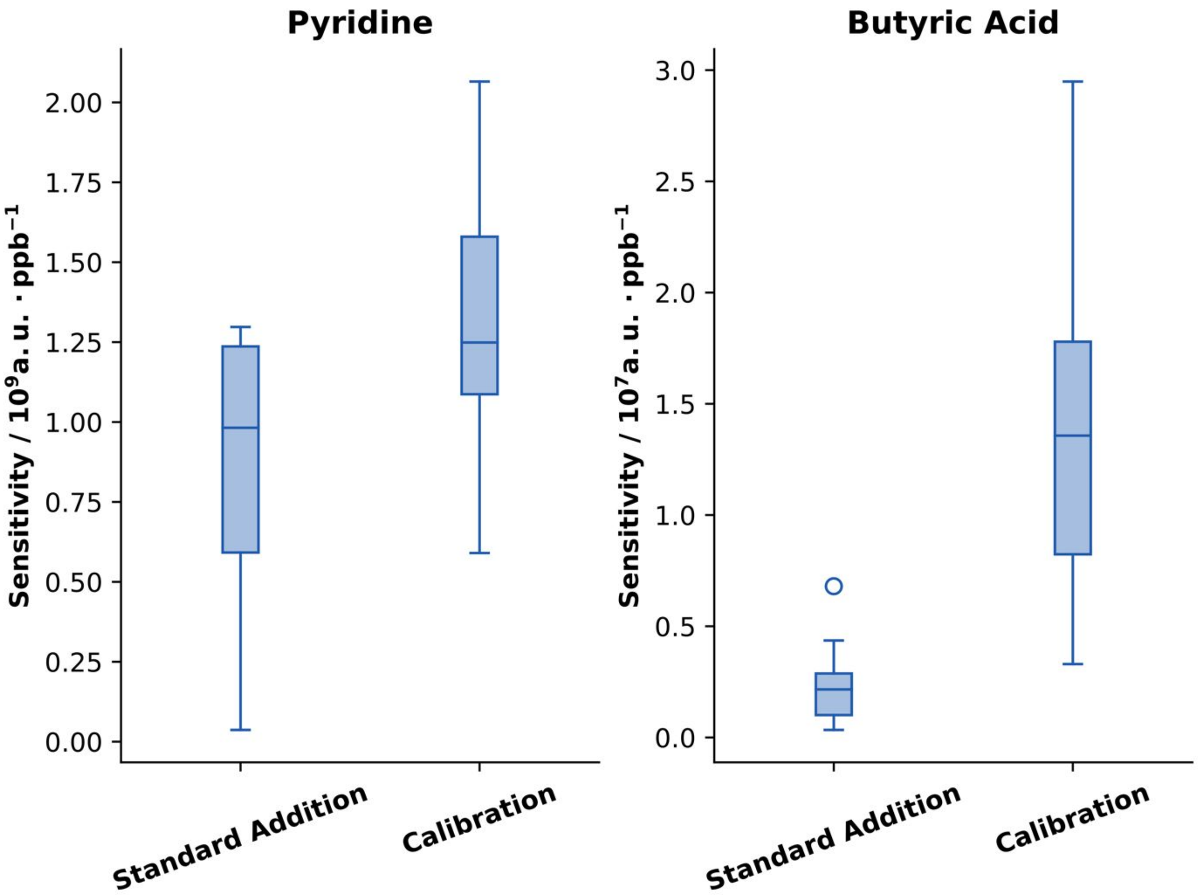

Figure S6. Boxplot of the obtained slopes of the standard addition and calibration curves. The median values are depicted as well as the 25th and 75th with the interquartile ranges and outliers. While for pyridine the slope values between calibration and standard addition differ to a slight degree, the values for butyric acid differ heavily.

References

(1) Lan, J.; Kaeslin, J.; Greter, G.; Zenobi, R. Minimizing Ion Competition Boosts Volatile Metabolome Coverage by Secondary Electrospray Ionization Orbitrap Mass Spectrometry. *Anal. Chim. Acta* **2021**, *1150*, 338209. https://doi.org/10.1016/j.aca.2021.338209.

(2) Sander, R. Compilation of Henry ' s Law Constants Discuss Version. *Atmos. Chem. and Phys. Discuss.* **2014**, No. 14, 29615–30521. https://doi.org/10.5194/acpd-14-29615-2014.
